# The role of the medical oncologist in the holistic care of patients with cancer in the Philippines

**DOI:** 10.3332/ecancer.2021.ed115

**Published:** 2021-08-27

**Authors:** Frederic Ivan Ting, Crizel Denise Uy, Katrina Gaelic Bebero, Danielle Benedict Sacdalan, Dennis Lee Sacdalan, Honey Sarita Abarquez, Arnold John Uson, Grace Nilo, Buenaventura Ramos

**Affiliations:** 1 Riverside Bacolod Cancer Care Center, Bacolod, 6100, Philippines; 2 San Juan De Dios Hospital, Manila, 1000, Philippines; 3 Franco Clinic and Hospital, Trento, 8505, Philippines; 4 University of the Philippines - Philippine General Hospital, Manila, 1000, Philippines; 5 Davao Doctors Hospital, Davao City, 8000, Philippines; 6 Perpetual Succour Hospital, Cebu City, 6000, Philippines; 7 St Luke's Medical Center - Global City, Manila, 1000, Philippines; 8 Cebu Doctors University Hospital, Cebu City, 6000, Philippines

**Keywords:** medical oncologist, role, cancer care, Philippines

## Abstract

In the past decade, cancer care in the Philippines has advanced in response to the complex heterogeneity inherent to the disease and the constantly increasing number of patients. Central to this development is the increased awareness and universal acceptance of the multidisciplinary team (MDT) approach in providing cancer care to optimize outcomes where medical oncologists play a vital role. This position paper summarizes the unique and multifaceted roles of the medical oncologist in ensuring that the best possible care is given to patients with cancer by actively participating in the whole spectrum of the patient’s journey—from diagnosis, treatment, supportive care, until providing palliative care and addressing end-of-life issues

Cancer is a complex and highly heterogenous disease that is best treated within the context of a multidisciplinary team (MDT) of highly qualified healthcare professionals [1, 5]. Central to the MDT is the role of the medical oncologist, who offers patients a comprehensive, systemic, and systematic approach to treatment and care which complements the skill sets of cancer specialists from other disciplines [6].

In response to the steadily increasing number of patients diagnosed with cancer and its increasing cost of treatment in the Philippines, the National Integrated Cancer Control Act (NICCA) (Philippines Republic Act 11215) was signed in February 2019 to strengthen cancer control in the country, increase cancer survivorship and reduce burden on families and cancer patients. At the heart of the law and critical to its implementation is the NICCA council which is in charge of crafting the implementing rules and regulations.

As the NICCA council is in the process of defining the roles of the different specialists, this position paper by the Philippine Society of Medical Oncology summarizes the essential contribution of medical oncology and the vital roles of medical oncologists in the holistic treatment of patients with cancer especially in the setting of a multidisciplinary approach.

## The medical oncologist is built on the highest standards of qualification

To become a medical oncologist in the Philippines, a physician has to complete three years of specialty training in internal medicine and thereafter pass the certifying examinations of the Philippine Specialty Board of Internal Medicine. This is followed by two to three years of subspecialty training in medical oncology. This training covers the entire gamut of the systemic treatment of cancer from initial evaluation, diagnosis, determination of appropriate, patient-tailored systemic therapy and its correct administration. This also includes the prevention and management of the adverse effects of treatment, which is a crucial element of cancer care. Finally, the medical oncologist is trained in post-treatment surveillance and the tenets of survivorship care. In short, medical oncologists are trained in the comprehensive care of patients living with cancer. Upon completion of their prescribed training, all medical oncologists are certified by the Philippine Specialty Board of Medical Oncology in one of the most rigorous subspecialty board examinations in the country. Even after certification, medical oncologists actively participate in continuing medical education (CME) programs to keep them up to date in the rapidly progressive and dynamic field of oncology. This is done to ensure that Filipinos living with cancer receive the highest standards of quality care [7, 8].

## The medical oncologist is a core member of the multidisciplinary team

The complex heterogeneity that characterizes the pathology of cancer has paved the way for the universal acceptance of the multidisciplinary team (MDT) approach in providing cancer care to optimize outcomes.

The core function of a multidisciplinary team (MDT) is to bring together a group of healthcare professionals from different subspecialties to determine the optimal treatment plan for the patient [9]. It is important to recognize that MDTs are longitudinal care structures that respond to current patient problems but at the same time, these have to be able to anticipate challenges that may develop during the continuum of care of a patient living with cancer. An effective MDT requires theoretical approaches that go beyond the more typical cross-sectional snapshots of team structure, process, and performance [10]. To fulfill this task, members of the MDT have to be uniquely qualified by training and are grounded in sound theory, which extends far beyond mere familiarity with general oncologic protocol and practice.

At the core of an MDT approach is the desire to provide quality patient-centered and patient specific care. The additional benefit of an MDT is the use of evidence-based decision-making, leading to the judicious use of limited resources, which maximizes benefits for both the individual patient and the health care system within which the MDT operates.

To this end, the experience of the Philippine General Hospital Polyp and Colorectal Cancer Study Group can serve to illustrate how a local MDT, with members performing clearly defined roles, can maximize patient outcomes. It has recently published its three-year data, which shows that survival outcomes of patients managed under the PhilHealth colorectal cancer Z-package program at the Philippine General Hospital is comparable to our regional neighbors and even to more developed (i.e., Western) countries [11]. This serves to highlight how the performance of defined roles among MDT members allows the provision of quality cancer care even within resource-constrained settings.

The medical oncologist is an essential member of the MDT who coordinates among the multi-specialty members of the team, integrating all the needs of the patients in the treatment plan. The composition of the MDT depends on the local availability of qualified healthcare professionals in a specific locality, but the contribution of the medical oncologist is essential to integrate all the information in the interest of the patient, regardless of the setting [6].

## The medical oncologist plays an essential role in improving cancer care

Our understanding of cancer has evolved from being a single, homogenous, and organ-based disease to that of a highly complex, heterogenous, and constantly changing group of molecularly diverse diseases. These diseases can occur simultaneously at different sites or evolve within the same organ [8]. This changing understanding has a significant impact on the practice of cancer care and has placed the medical oncologist in a core position to provide personalized care to patients who are most likely to benefit from a specific intervention while sparing them from unnecessary toxicity. Furthermore, the medical oncologist actively participates in the whole spectrum of the patient’s journey—from diagnosis, treatment, supportive care, until providing palliative care and addressing end-of-life issues.

**Figure 1.**
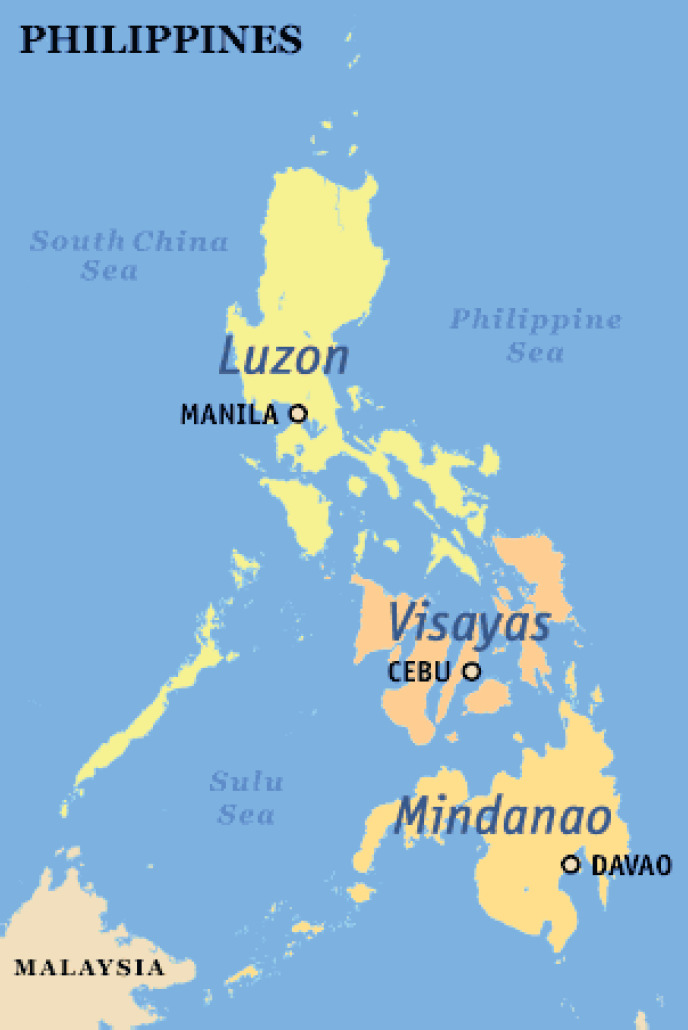
Map of the Philippines showing the location of the different training programs for Medical Oncology—Manila, Cebu, and Davao.

To provide comprehensive and safe services to an increasing number of patients, the Philippine Society of Medical Oncology (PSMO) has taken large strides in producing more subspecialists without compromising quality. More hospitals across the country now serve as training institutions for internists who wish to subspecialize in this field. To ensure optimal geographical distribution of medical oncologists, the newest training programs of the PSMO have started in Cebu (Visayas) and Davao (Mindanao) (Figure 1).

## Medical oncologists are leaders in the treatment of patients with cancer

Founded in 1969, the PSMO is a professional non-profit organization of board-certified Medical Oncologists that aims to advance the science, the ethical, and the holistic practice of cancer care in the Philippines. It continually strives to be an internationally recognized organization of competent and compassionate medical oncologists inspired and committed to integrate a multidisciplinary approach to the optimal care of the Filipino patient living with cancer.

The Society undertakes research to foster the development and advancement of oncology. It provides opportunities for continuing medical education and further professional development of its members. PSMO educates the public at large and allied medical specialties in the holistic approach to the care of patients living with cancer and their families. It nurtures fellowship and encourages openness and cooperation among its members as it actively pursues the standards of excellence in the science and practice of Oncology.

## Conclusion

The PSMO envisions the Philippines as a country wherein every Filipino living with cancer can receive optimal cancer care by expanding its accessibility, availability, and affordability [12]. It aims to achieve these by providing expertise in newly opened cancer centers, offering fellowship training programs that meet both national and international standards, providing opportunities for patients to be enrolled in high quality and relevant clinic trials, and championing multidisciplinary team care towards the best outcomes and quality of life for patients living with cancer.

## Funding declaration

This paper did not receive any funding.

## Conflicts of interest

The authors have no conflicts of interest to declare.
